# The association of tumor-to-background ratios and SUVmax deviations related to point spread function and time-of-flight F18-FDG-PET/CT reconstruction in colorectal liver metastases

**DOI:** 10.1186/s13550-015-0111-5

**Published:** 2015-05-06

**Authors:** Julian MM Rogasch, Ingo G Steffen, Frank Hofheinz, Oliver S Großer, Christian Furth, Konrad Mohnike, Peter Hass, Mathias Walke, Ivayla Apostolova, Holger Amthauer

**Affiliations:** Klinik für Radiologie und Nuklearmedizin, Universitätsklinikum Magdeburg A.ö.R., Otto-von-Guericke Universität Magdeburg, Leipziger Straße 44, Magdeburg, 39120 Germany; Helmholtz-Zentrum Dresden-Rossendorf, PET Center, Institute of Radiopharmaceutical Cancer Research, Bautzner Landstraße 400, Dresden, 01328 Germany; Klinik für Strahlentherapie, Universitätsklinikum Magdeburg A.ö.R., Otto-von-Guericke Universität Magdeburg, Leipziger Straße 44, Magdeburg, 39120 Germany

**Keywords:** F18-FDG-PET/CT, SUVmax, Reconstruction algorithm, PSF, TOF, Tumor-to-background ratio, Colorectal liver metastases, Target volume definition

## Abstract

**Background:**

The maximum standardized uptake value (SUVmax) is a common clinical parameter for quantification in F18-fluorodeoxyglucose positron emission tomography/computed tomography (FDG-PET/CT), but it is influenced by image reconstruction. The aim of this study was to analyze the association of SUVmax deviations related to point spread function (PSF) and time-of-flight (TOF) reconstruction with tumor-to-background ratios (TBR) in colorectal liver metastases (CRLM).

**Methods:**

Fifteen patients (f, 6; m, 9; median age, 59 years; range, 32 to 72 years) with 28 liver metastases were included retrospectively. FDG-PET/CT imaging (median activity, 237 MBq; range, 231 to 252 MBq; median uptake, 61 min; range, 55 to 67 min) was performed on a Siemens Biograph mCT 64 followed by image reconstruction using 3D-ordered subset expectation maximization (3D-OSEM) or 3D-OSEM with PSF modeling - both with and without TOF information. Differences in SUVmax were analyzed using the Friedman test and Wilcoxon test for paired non-parametric data. The correlation of inter-method differences with the lesions’ TBR was studied using Spearman’s rank correlation coefficient (rho). Differences between lesions with low (<4.8) and high (>4.8) TBR were analyzed using the Mann-Whitney *U* test (TBR measured with 3D-OSEM; binarized by its median).

**Results:**

There was a significant correlation of the lesions’ TBR with relative SUVmax differences related to PSF (PSF + TOF vs. 3D-OSEM + TOF, rho = 0.61; PSF vs. 3D-OSEM, rho = 0.52) or TOF (PSF + TOF vs. PSF, rho = −0.58; 3D-OSEM + TOF vs. 3D-OSEM, rho = −0.61). Accordingly, PSF algorithms only showed higher SUVmax than non-PSF algorithms in lesions with a high TBR (median differences at low/high TBR, +2.6%/+9.1% [PSF + TOF vs. 3D-OSEM + TOF]; +0.7%/+6.4% [PSF vs. 3D-OSEM]). TOF integration also led to higher SUVmax but mainly at low TBR (low/high TBR, +10.4%/+1.8% [PSF + TOF vs. PSF]; +8.6%/−0.1% [3D-OSEM + TOF vs. 3D-OSEM]).

**Conclusions:**

Both PSF and TOF reconstruction resulted in a substantial alteration of SUVmax in CRLM. TOF provided the highest SUVmax increase in low-contrast lesions while - vice versa - PSF showed the most relevant increase in high-contrast lesions. Thus, one should be aware that quantitative analyses of lesions with varying TBR, e.g., in radiotherapy or follow-up studies, may be mainly affected by either PSF or TOF reconstruction, respectively.

## Background

Combined F18-fluorodeoxyglucose positron emission tomography/computed tomography (FDG-PET/CT) has proven its significant impact on the therapeutic management in patients with colorectal liver metastases (CRLM) when compared to conventional imaging methods, such as CT or magnetic resonance imaging (MRI) [1-4]. Moreover, the maximum standardized uptake value (SUVmax) as a common quantitative measure of the focal FDG uptake may be helpful for therapy response assessment [5] or as a basis for target volume definition in radiotherapy planning [6,7].

However, FDG-PET quantification is influenced by the reconstruction algorithm used [8,9]. Recent algorithms feature iterative calculations with integration of time-of-flight (TOF) analysis (to approximate the real location of the positron-electron annihilation) and the point spread functions (PSF) of the PET scanner to account for its specific detection properties. Prieto et al. [10] and Knäusl et al. [11,12] reported higher SUV and smaller metabolic tumor volumes (MTV) when applying these algorithms compared to common ordered subset expectation maximization (OSEM) algorithms. Accordingly, they should be used with caution for the purpose of quantification. Although a reliable reference value is missing in clinical lesions, the assumed SUVmax overestimation by PSF-based algorithms could result in a distorted assessment of therapy response or inaccurate volume definition.

In a recent publication, we showed based on phantom measurements that the mentioned inter-method differences depend on the respective signal-to-background ratio (SBR) and are nearly absent at low SBR which are typical for hepatic lesions [13]. Furthermore, an independent evaluation of TOF-related effects on quantification in liver lesions has not been performed so far. Thus, the aim of the present study was to evaluate the influence of PSF and TOF reconstruction and tumor-to-background ratios (TBR) on SUVmax in patients with CRLM.

## Methods

### Patients

This retrospective, explorative single-center study included 15 patients [female, *n* = 6; male, *n* = 9; median age, 59 years (range, 32 to 72 years)] which had been referred for FDG-PET/CT in our department to evaluate potential palliative treatment options including systemic chemotherapy and/or local ablative therapy. All patients suffered from FDG-avid liver metastases from colorectal cancer (rectal cancer, *n* = 9; colon cancer, *n* = 6), and all patients had undergone at least one line of systemic chemotherapy (median, two lines; range, one to five lines). Written informed consent of the patients was obtained for the publication of this report and any accompanying images. This analysis was performed in compliance with the Helsinki Declaration and approved by the ethics commission of the Otto-von-Guericke University Magdeburg (ID number: RAD196).

### PET/CT

PET/CT imaging was performed using the tracer F18-FDG and a dedicated PET/CT device (Biograph mCT 64®; Siemens Healthcare, Erlangen, Germany) according to procedure guidelines for tumor PET imaging by the European Association of Nuclear Medicine [14]. Whole-body imaging was performed from the vertex to the proximal femora within six to eight bed positions (emission, 3 min each) and axial bed coverage of 216 mm each (Siemens TrueV®; bed overlap, 89 mm). A median activity of 237 MBq (range, 231 to 252 MBq) was applied intravenously with a median uptake time of 61 min (range, 55 to 67 min). A low-dose CT was used for attenuation correction and anatomical mapping (50 mA, 120 kV, 0.5 s/rotation; pitch, 0.8).

### Image reconstruction

FDG-PET raw data were reconstructed with four algorithms and respective presets provided by the manufacturer: 3D-ordered subset expectation maximization (3D-OSEM; iterations, 2; subsets, 24), 3D-OSEM + time-of-flight analysis (3D-OSEM + TOF; iterations, 2; subsets, 21), iterative reconstruction with system-specific PSF modeling (PSF; Siemens TrueX®, ‘HD∙PET’; iterations, 2; subsets, 24), and PSF + TOF (Siemens ‘ultraHD∙PET’; iterations, 2; subsets, 21) [15]. The projection data were reconstructed with 3-mm slice thickness (rows, 200; columns, 200; voxel size, 4.1 × 4.1 × 3.0 mm). After reconstruction, a Gaussian filter (FWHM, 2 mm) was applied to all data. Attenuation correction CT raw data were reconstructed with a slice thickness of 3 mm and a filter for abdominal low-dose CT (convolution kernel, B30f).

### SUV and TBR

All SUV were measured using dedicated software (ROVER, version 2.1.4, ABX advanced biochemical compounds GmbH, Radeberg, Germany). The TBR was defined as the ratio of the lesions’ SUVmax and the SUVmean of healthy liver tissue (background) determined for each reconstruction algorithm. The background SUVmean was measured within a spherical volume of interest (diameter, 50 mm) positioned in an area of physiological liver tissue.

### Dedicated CECT and MRI

The morphological lesion volume (expressed in ml) was measured in MRI data if available (seven patients) or in contrast-enhanced CT (CECT) data (eight patients) by manual delineation using ROVER software. CECT or MRI was performed within 8 weeks of the FDG-PET/CT examination (median, 1 day; IQR, 0 to 14 days; range, 0 to 48 days). CECT data were acquired 70 s after intravenous injection of 80 to 150 ml of a non-ionic iodinated contrast agent (Imeron 300, iomeprol 300, Bracco ALTANA Pharma GmbH, Konstanz, Germany). CT scans were performed from the apex of the lungs to the thigh (automatic tube current modulation with maximum tube current, 230 mAs; tube voltage, 120 kV; gantry rotation, 0.5 s). MRI of the liver was performed using a 1.5-T Philips Acheiva® (Philips, Best, The Netherlands) in enhanced T1 High Resolution Isotropic Volume Excitation (eTHRIVE) mode after intravenous administration of 0.025 mmol/kg bodyweight Gd-EOB-DTPA (Primovist®, Bayer, Leverkusen, Germany).

### Statistical analysis

Data analyses were carried out using SPSS 22 (IBM Corporation, Armonk, NY, USA) and R 3.1.3 (Foundation for Statistical Computing, Vienna, Austria, 2015, http://www.R-project.org). Due to a small sample size, non-parametric distribution of data was assumed. Descriptive parameters were expressed as median, interquartile range (IQR), and range and depicted as box plots. SUVmax differences between reconstruction algorithms were investigated using the Friedman test and Wilcoxon signed-rank test for paired non-parametric data. The correlation between relative SUVmax differences and the lesions’ TBR was analyzed using the Spearman’s rank correlation coefficient. Furthermore, a binarization of the measured TBR was performed based on its median value measured in 3D-OSEM reconstructed data (<4.8/>4.8) followed by the Mann-Whitney *U* test to compare SUVmax differences in lesions classified by low and high TBR, respectively. Similarly, the lesion volumes were binarized by the median value (9.3 ml) to compare small and large lesions. General linear models (GLM) including the TBR measured in 3D-OSEM reconstructed data, the lesion volume, and their interaction (TBR * lesion volume) were calculated to examine the association of both variables with the SUVmax differences. Corresponding interaction plots based on binarized TBR and lesion volume were created. Statistical significance was assumed at a *P* value of less than 0.05.

## Results

Twenty-eight FDG-avid hepatic target lesions were analyzed. The median background SUVmean was similar for all reconstruction algorithms (median SUVmean, 2.8; range, 2.0 to 4.1). The measured SUVmax and calculated TBR are summarized in Table 1. Inter-method SUVmax differences and their correlation with the TBR were assessed separately for PSF and TOF. Accordingly, the following section provides a comparison of corresponding PSF and non-PSF algorithms (PSF + TOF vs. 3D-OSEM + TOF; PSF vs. 3D-OSEM) or corresponding TOF and non-TOF algorithms (PSF + TOF vs. PSF; 3D-OSEM + TOF vs. 3D-OSEM), respectively.

**Table 1:** **SUVmax and TBR for each reconstruction algorithm**

	**PSF + TOF**	**PSF**	**3D-OSEM + TOF**	**3D-OSEM**
SUVmax				
Median	12.2	11.0	11.6	11.1
IQR	10.5 to 17.4	9.3 to 17.2	9.9 to 16.0	9.4 to 16.8
Range	5.5 to 47.1	4.4 to 47.5	5.3 to 42.6	4.8 to 43.8
TBR				
Median	5.0	4.7	4.6	4.8
IQR	3.3 to 6.3	3.2 to 6.1	3.4 to 5.8	3.1 to 5.6
Range	1.8 to 16.9	1.7 to 16.5	1.9 to 15.3	1.8 to 15.3

Median values of SUVmax and TBR displayed for all reconstruction algorithms with their respective IQR and range.

### SUVmax differences between PSF and non-PSF algorithms

In lesions with low TBR (<4.8), PSF + TOF provided comparable SUVmax as 3D-OSEM + TOF (median difference, +2.6%; *P* = 0.1). PSF featured similar SUVmax as 3D-OSEM (+0.7%; *P* = 0.73). In lesions with high TBR (>4.8), PSF + TOF showed significantly higher SUVmax than 3D-OSEM + TOF (median difference, +9.1%; *P* < 0.01). PSF also provided higher SUVmax than 3D-OSEM (+6.4%; *P* < 0.01).

**Figure 1:**
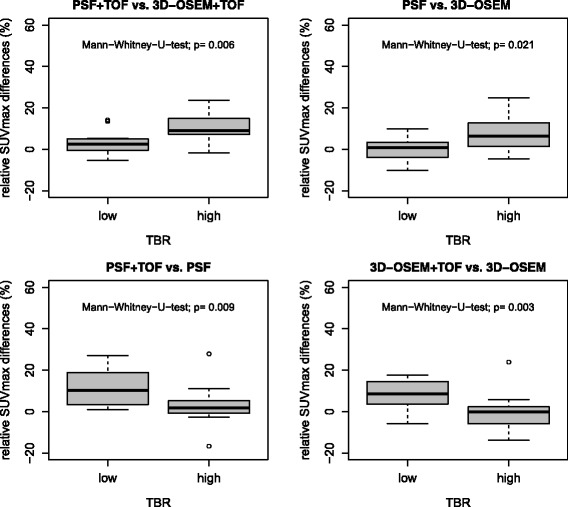
Relative SUVmax differences between reconstruction algorithms. Box plots of relative SUVmax differences in lesions with low TBR and high TBR. Comparison of PSF vs. non-PSF (upper row) as well as TOF vs. non-TOF algorithms (lower row). Outliers are marked as circles.

**Figure 2:**
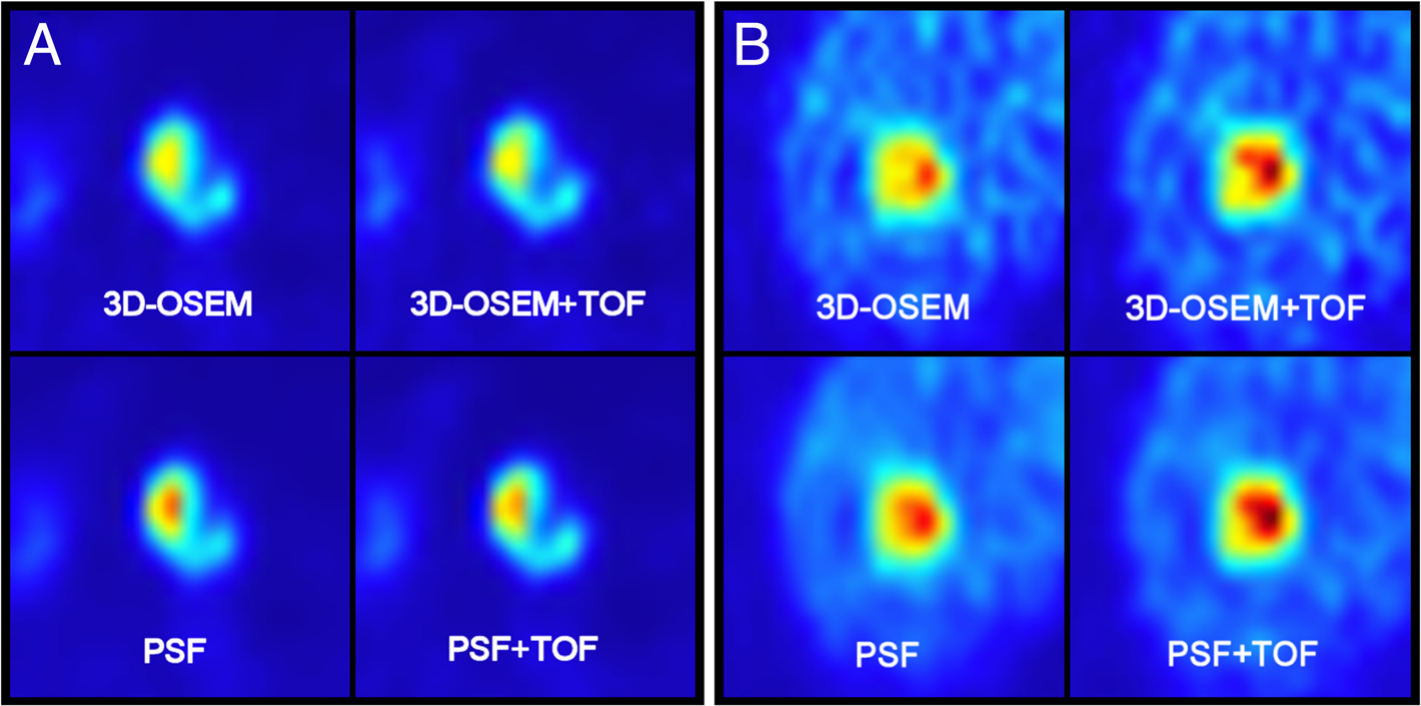
Representative examples of lesions with high and low TBR. This figure displays the axial FDG-PET images (jet color table) of a lesion with high TBR (**A**; TBR, 11.4; volume, 10.0 ml) and a lesion with low TBR (**B**; TBR, 3.6; volume, 7.9 ml) for all analyzed reconstruction algorithms. The windowing level was the same for all differently reconstructed data (but different between A and B). At high contrast (A), the SUVmax is mainly increased by PSF integration (+22% and +25%) and marginally affected by TOF (+0.2% and +2%). At low contrast (B), the opposite is true (PSF, +3% and +4%; TOF, +15% and +16%).

The inter-method differences for PSF + TOF vs. 3D-OSEM + TOF and PSF vs. 3D-OSEM were significantly higher in high-contrast lesions compared to low-contrast lesions (Mann-Whitney *U* test; each *P* < 0.05). All differences are also displayed in Table 2, while Figure 1 displays corresponding box plots. Figure 2 provides axial PET images of representative examples to illustrate the effect of the TBR on the extent of PSF-related SUVmax differences.

**Table 2:** **Relative SUVmax differences - PSF vs. non-PSF**

**Difference**	**PSF + TOF vs. 3D-OSEM + TOF**		**PSF vs. 3D-OSEM**	
	**Low TBR**	**High TBR**	**Low TBR**	**High TBR**
	**n.s.**	**	**n.s.**	**
Median	2.6	9.1	0.7	6.4
IQR	−1.2 to 5.0	6.0 to 15.2	−4.0 to 3.4	0.9 to 12.9
Range	−5.3 to 14.2	−1.6 to 23.7	−10.1 to 9.8	−4.7 to 24.8

Median, IQR, and range of relative SUVmax differences between PSF and non-PSF algorithms displayed separately for low (<4.8) and high (>4.8) TBR. Wilcoxon test: n.s., not significant; **P < 0.01.

### SUVmax differences between TOF and non-TOF algorithms

In lesions with low TBR (<4.8), PSF + TOF provided significantly higher SUVmax in comparison with PSF (median difference, +10.4%; *P* < 0.01). 3D-OSEM + TOF also featured higher SUVmax than 3D-OSEM (+8.6%; *P* < 0.01). In lesions with high TBR (>4.8), PSF + TOF showed comparable SUVmax in comparison with PSF (median difference, +1.8%; *P* = 0.2). 3D-OSEM + TOF featured similar SUVmax as 3D-OSEM (−0.1%; *P* = 0.73).

The SUVmax differences for PSF + TOF vs. PSF and 3D-OSEM + TOF vs. 3D-OSEM were significantly higher at low TBR compared to high TBR (each *P* < 0.01). All differences are also displayed in Table 3. Figure 1 provides corresponding box plots and Figure 2 illustrates representative examples.

**Table 3:** **Relative SUVmax differences - TOF vs. non-TOF**

**Difference**	**PSF + TOF vs. PSF**		**3D-OSEM + TOF vs. 3D-OSEM**	
	**Low TBR**	**High TBR**	**Low TBR**	**High TBR**
	**	**n.s.**	**	**n.s.**
Median	10.4	1.8	8.6	−0.1
IQR	3.1 to 19.0	−0.8 to 5.5	3.3 to 14.8	−6.0 to 2.4
Range	1.0 to 27.2	−16.7 to 27.9	−5.9 to 17.5	−13.9 to 24.0

Median, IQR, and range of relative SUVmax differences between TOF and non-TOF algorithms displayed separately for low (<4.8) and high (>4.8) TBR. Wilcoxon test: n.s., not significant; **P < 0.01.

### Correlation of relative SUVmax differences with the TBR

The relative SUVmax differences between PSF + TOF and 3D-OSEM + TOF were positively correlated with the lesions’ TBR measured for 3D-OSEM (*ρ* = 0.6; *P* < 0.01). Differences between PSF and 3D-OSEM also showed a significant positive correlation with the respective TBR (*ρ* = 0.52; *P* < 0.01).

**Figure 3:**
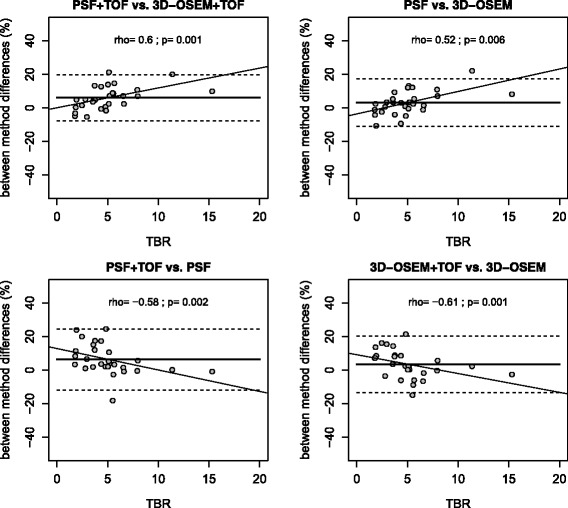
Correlation of relative SUVmax differences with the TBR. Correlation plots of relative SUVmax differences and the lesions’ TBR between corresponding PSF and non-PSF algorithms (upper row) or TOF and non-TOF algorithms (lower row), respectively. The solid and dashed lines represent mean ± two standard deviations.

Conversely, the relative SUVmax differences between PSF + TOF and PSF were negatively correlated with the lesions’ TBR (*ρ* = −0.58; *P* < 0.01). Differences between 3D-OSEM + TOF and 3D-OSEM featured a negative correlation (*ρ* = −0.61; *P* < 0.01) with the TBR, too (Table 4, Figure 3). There was no significant correlation of SUVmax differences between PSF + TOF and 3D-OSEM with the TBR (*ρ* = −0.23; *P* = 0.269).

**Table 4:** **Correlation between relative SUVmax differences and the TBR**

**vs.**	**PSF**	**3D-OSEM + TOF**	**3D-OSEM**
PSF + TOF	*ρ* = −0.58	*ρ* = 0.6	*ρ* = −0.23
	*P = 0.002*	*P = 0.001*	*P* = 0.269
PSF	–	*ρ* = 0.68	*ρ* = 0.52
		*P < 0.001*	*P = 0.006*
3D-OSEM + TOF	–	–	*ρ* = −0.61
			*P = 0.001*

Spearman’s rho (*ρ*) and degree of significance for the correlation of relative SUVmax differences related to PSF or TOF with the lesions’ TBR. Significant results are printed in italics.

### Dependency of relative SUVmax differences on the lesion volume

The lesion volume measured in CECT or MRI data ranged from 0.8 to 60.3 ml (median, 9.3 ml; IQR, 2.8 to 29.3 ml). There was a significant positive correlation of the lesion volume and the TBR measured in 3D-OSEM reconstructed data (*ρ* = 0.56; *P* < 0.01). Lesions with a high TBR were significantly larger (median, 18.5 ml; IQR, 8.1 to 45.1 ml) than lesions with low TBR (median, 3.7 ml; IQR, 2.1 to 14.1 ml; *P* < 0.01).

There was no significant correlation between PSF-related SUVmax differences and the lesion volume (PSF + TOF vs. 3D-OSEM + TOF, *ρ* = 0.03; *P* = 0.88; PSF vs. 3D-OSEM, *ρ* = 0.08; *P* = 0.68). SUVmax differences between TOF and non-TOF algorithms showed a significant negative correlation with the lesion volume (PSF + TOF vs. PSF, *ρ* = −0.5; *P* < 0.01; 3D-OSEM + TOF vs. 3D-OSEM, *ρ* = −0.41; *P* < 0.05). Neither PSF-related nor TOF-related SUVmax differences varied significantly between small and large lesions (each *P* > 0.05).

**Figure 4:**
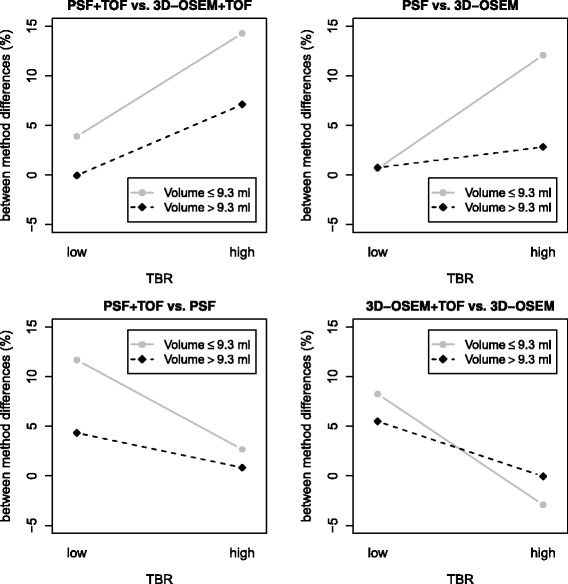
Interaction plots between the TBR and the lesion volume. Interaction plots of the lesions’ TBR and the lesion volume on relative SUVmax differences between corresponding PSF and non-PSF algorithms (upper row) or TOF and non-TOF algorithms (lower row), respectively. The TBR and the lesion volume were binarized by their respective median value. In general, an increased effect of the TBR on SUVmax differences is observed in smaller lesions. This interaction between TBR and lesion volume is indicated by non-parallel courses of corresponding gray and black dashed lines.

The GLM showed a significant association of SUVmax differences between PSF and non-PSF algorithms with the TBR (PSF + TOF vs. 3D-OSEM + TOF, *P* < 0.001; PSF vs. 3D-OSEM, *P* < 0.001) but not with the lesion volume (both *P* = 0.91). The interaction between the TBR and lesion volume was significant (both *P* < 0.05; Figure 4). TOF-related SUVmax differences were associated with the TBR (PSF + TOF vs. PSF, *P* < 0.05) or showed a tendency towards an association (3D-OSEM + TOF vs. 3D-OSEM, *P* = 0.054). There was no significant association with the lesion volume (*P* = 0.2 and *P* = 0.18). The interaction between TBR and lesion volume was also not significant (both *P* > 0.15; Figure 4).

## Discussion

The present study focused on SUVmax in patients with CRLM, examining the influence of PSF and TOF reconstruction algorithms as well as different TBR.

The integration of PSF and TOF into reconstruction revealed relevant impact on SUVmax when lesions were separated by their TBR (low, <4.8; high, >4.8) measured in 3D-OSEM reconstructed data. In lesions with a high TBR, both PSF + TOF and PSF showed significantly higher SUVmax compared to the corresponding non-PSF algorithms. The median relative differences were slightly higher for combined PSF + TOF (PSF + TOF vs. 3D-OSEM + TOF, +9.1%) than for PSF alone (PSF vs. 3D-OSEM, +6.4%). Of course, these results are, strictly speaking, only valid for the specific scanner and reconstruction software used in this study, but it can be expected that with other systems, similar SUV deviations would occur.

Knäusl et al. evaluated nine lung lesions regarding SUVmax based on PSF (also Siemens TrueX® algorithm) and OSEM reconstruction and reported even higher differences of 19% on average [12]. This may be due to typically higher TBR in pulmonary lesions with pronounced SUV differences and due to an increased noise level caused by the higher product of iterations and subsets used by Knäusl et al. (4 iterations, 21 subsets). Akamatsu et al. analyzed 41 lymph node metastases and observed SUVmax differences of about +40% between PSF + TOF and OSEM + TOF as well as between PSF and OSEM. Again, differences increased with an increasing number of iterations [16].

However, the observed inter-method differences related to PSF were significantly correlated with the lesions’ TBR and were significantly lower in lesions with a low TBR. A recent study on FDG-PET phantom measurements analyzed radial activity concentration profiles of spheres filled with a solution of F18-FDG at three different SBR. It demonstrated that signal elevations at the spheres’ boundaries (known as *Gibbs* artifacts [17]) occurred only in PSF + TOF and PSF and only at medium (6:1) and high (16:1) SBR. In analogy to the current results, this led to an artificially increased SBR and to the observed deviations in quantitative parameters [13].

The integration of TOF analysis resulted in SUVmax differences comparable to those observed for additional PSF (PSF + TOF vs. PSF, +10.4%; 3D-OSEM + TOF vs. 3D-OSEM, +8.6%). However, these deviations were measured in lesions with a low TBR while SUV in high-contrast lesions were significantly less affected by TOF. These inverse findings of PSF and TOF may explain why no correlation was observed for differences between combined PSF + TOF and 3D-OSEM (non-PSF, non-TOF) and the TBR. In other words, PSF + TOF increased SUVmax across the entire range of the TBR when compared to 3D-OSEM (correlation plot not shown). As a consequence, a differentiated assessment of PSF + TOF and PSF with regard to varying TBR is required. There are scarce data on the independent influence of TOF analysis on SUV in clinical lesions. In the abovementioned study, Akamatsu et al. reported SUVmax differences of only +2% between PSF + TOF and PSF as well as between OSEM + TOF and OSEM, probably due to relatively high TBR of the analyzed lesions. Nevertheless, a TOF-related SUVmax increase was mainly observed in lesions with low SUVmax [16]. Schaefferkoetter et al. assessed the signal-to-noise ratios (SNR) of FDG-avid foci which were artificially added to FDG-PET scans of 23 patients. The authors reported increasing SNR of OSEM + TOF, PSF, and PSF + TOF compared to OSEM. In analogy to the current results, the relative SNR gain of OSEM + TOF vs. OSEM was higher in lesions with lower count rate (20,000 vs. 60,000 counts) [18]. Taniguchi et al. performed phantom measurements (lesion diameter, 10 to 37 mm) and clinical studies in hepatic lesions (average diameter, 10.7 mm) to analyze the influence of PSF and TOF on lesion contrast, coefficient of variation (CV) of the background activity, as well as lesion SNR. Both PSF and TOF independently increased liver lesion SNR with the best tradeoff between lesion contrast and CV for combined PSF + TOF. Furthermore, PSF + TOF reduced the CV of the liver in overweight patients (>70 kg) to the CV level in OSEM reconstructed data of normal-weight patients (<70 kg) [19]. That such differences in quantitative measures can also result in improved lesion detection was shown by El Fakhri et al. who analyzed the influence of TOF integration on detection rates in simulated liver and lung lesions. TOF improved lesion detection over non-TOF PET in both hepatic and pulmonary lesions but showed the greatest advantage at low contrast (contrast, 2.0:1 vs. 5.7:1) and in patients with higher body mass index (BMI; >30 vs. <30) [20].

It is well known that the TBR is affected by the lesion size due to partial volume effects which are more pronounced in smaller lesions [21,22]. As we observed such a positive correlation between the lesion volume and the TBR, we included the TBR, the lesion volume, and their interaction into GLMs. For PSF integration, the GLM showed no association between SUVmax differences and the lesion size whereas a highly significant association with the TBR was observed. In addition, there was a significant interaction between the TBR and the lesion volume, indicating that the effect of the TBR on PSF-related SUVmax differences depends on the lesion volume. Thus, we observed an increased impact of the TBR in smaller lesions. Also, in TOF integration, the SUVmax differences showed a significant association with the TBR (PSF + TOF vs. PSF) or a tendency towards an association (3D-OSEM + TOF vs. 3D-OSEM). In comparison to PSF, a similar effect of the lesion volume can be observed in the interaction plots. However, in GLM, the interaction term showed no significance which may be caused by a high variance of SUVmax differences in combination with a small sample size. This dependency of TOF-related effects on the lesion volume is in agreement with previous studies showing that the impact of TOF is especially relevant in small lesions [23,24].

SUVmax are commonly used for threshold-dependent volume definition in a clinical setting. If the delineation is strictly based on SUVmax (i.e., relative threshold without background correction), these differences would also implicate corresponding MTV deviations as reported previously [12]. However, we refrained from volumetric analyses as such delineation methods may not reflect the clinical practice where more sophisticated algorithms with background correction or manual MTV delineation are required - especially in hepatic lesions [14,25,26]. Thus, the actual MTV deviations may be lower and less dependent on the TBR than the current results on SUVmax deviations suggest.

Nevertheless, these results underline that quantitative analyses in radiotherapy planning, follow-up, or multicenter studies can be distorted not only by different reconstruction settings but also when comparing lesions located in different organs (e.g., lung and liver) or one lesion with varying TBR measured over the course of time. Depending on the lesions’ TBR, one must be aware of SUVmax deviations mainly caused by PSF or TOF, respectively. This may be particularly true for hepatic lesions that feature a range of TBR in which both PSF- and TOF-related effects are relevant.

The present study is limited by the retrospective inclusion of only 15 patients with 28 lesions which may impair an accurate interpretation of the data that were characterized by relatively large IQRs.

## Conclusions

Both PSF and TOF integration independently resulted in substantially increased SUVmax in CRLM. However, PSF- and TOF-related deviations showed an inverse correlation with the lesions’ TBR. TOF showed the highest SUVmax deviations in low-contrast lesions, whereas PSF revealed a substantial effect on SUVmax in high-contrast lesions. Although an interaction between the TBR and lesion volume was observed, these effects were still present after adjustment for the lesion volume. Thus, quantitative analyses in radiotherapy planning, follow-up, or multicenter studies can be distorted when comparing lesions located in different organs or one lesion with varying TBR measured over the course of time.
